# Deep Inferior Epigastric Perforator Flap for Immediate Breast Reconstruction following Mastectomy in Breast Cancer Patients: An Initial Experience in Vietnam

**DOI:** 10.1155/2023/5964040

**Published:** 2023-01-10

**Authors:** Xuan Hau Nguyen, Viet Dung Pham Thi, Xuan Hien Nguyen, Thi Dung Vu, Hop Nhan Nguyen, Quang Dao Pham, Van Ty Ngo

**Affiliations:** ^1^Hanoi Medical University, Vietnam; ^2^Oncology and Palliative Care Department, Hanoi Medical University Hospital, Vietnam; ^3^Aesthetic Plastic Surgery Department, Hanoi Medical University Hospital, Vietnam

## Abstract

**Background:**

Breast reconstruction in breast cancer patients is an optional surgery that improves the quality of life while preserving the efficacy of chemotherapy and radiotherapy. Deep inferior epigastric perforator (DIEP) flap is a new but reliable and safe technique for autologous breast reconstruction. After mastectomy, immediate reconstruction is the preferred method because of its aesthetic result and convenience. This study is aimed at summarizing our experience in DIEP flap for immediate breast reconstruction.

**Methods:**

A prospective study was performed on 30 breast cancer patients who underwent intermediate breast reconstruction for DIEP flap after mastectomy from June 2019 to June 2021 in Hanoi Medical University Hospital. Clinicopathology characteristics, tumor stage, treatment, and complications were evaluated.

**Result:**

The mean age of patients was 44.9 (range: 29-73 years). 86.7% of patients were in stages I and II. Five patients (16.7%) received neoadjuvant chemotherapy. 20 patients (66.7%) underwent nipple-sparing mastectomy (NSM) procedures. The mean operating time was 341 minutes. The mean time to receive chemotherapy was 34.68 days. The mean number of perforators was 1.30. The overall flap success rate was 90%. Twelve patients (40%) experienced complications. Four patients (13.3%) returned to the operating room due to venous congestions. Two patients (6.67%) had complete flap loss. Other complication: fat necrosis (6.7%), seroma (13.3%), partial flap loss (3.3%), abdominal wound dehiscence (6.7%), pneumonia (3.3%), and pulmonary embolism (6.7%). After one-month postoperation, 88.9% of patients were satisfied with their breasts, and 74.07% were satisfied with the operation.

**Conclusion:**

DIEP flap is a new but reliable and safe technique for autologous breast reconstruction. Though patients opting for breast reconstruction still have a low risk of complication and reconstruction failure, this procedure should be used more frequently in appropriate patients to improve their quality of life.

## 1. Introduction

According to GLOBOCAN 2020, female breast cancer is the most common cancer, with an estimated 2.3 million new cases (11.7%); however, the mortality has decreased considerably to the 5th leading cause of mortality due to advances in diagnosis and treatment [1]. Because 5-year survival rates for early-stage breast cancer are relatively high, the effects of breast cancer and its treatment on quality of life become more important, which may affect the decision-making process [2].

In general, patients with breast cancer undergo primary surgery (lumpectomy or mastectomy) and regional lymphadenectomy. Breast-conserving therapy followed by radiation therapy is an alternative to mastectomy, but tumor-to-breast volume ratio is an important criterion associated with the cosmetic result. Patients with small breasts would have less impressive cosmetic results and lower quality of life than others.

In Vietnam, mastectomy is still being applied in most cases. After this surgery, patients may feel less attractive, unhappy with their scars, and sadly, even refuse adjuvant therapy due to depression [3].

Breast reconstruction has been split into categories based on its material and timing surgery: implant reconstruction or autologous tissue flaps, immediately or months after mastectomy. Of which, in Vietnam, the DIEP flap is a new but reliable and safe technique for autologous breast reconstruction. Systematic reviews and prospective studies showed that patients reconstructed by DIEP flap are satisfied with cosmetic results, body shape and breasts, physical activity, sexuality, and psychological health [4]. In addition, studies have shown that mastectomy combined with plastic surgery is as safe as mastectomy alone in terms of local recurrence rate, disease-free survival, and overall survival [5, 6].

Regarding the timing of reconstruction, the immediate procedure is the preferred method because of its aesthetic result and convenience in implementation [7]. DIEP flap was reported for the first time in 1994. This flap offers the same advantages as the TRAM flap, but the donor site has few complications by preserving rectus muscle [8]. Thus, DIEP flap immediate breast reconstruction has been the gold standard for breast reconstruction in many oncology centers [9].

This study is aimed at evaluating the initial result of 30 breast cancer patients who underwent mastectomy and immediate breast reconstruction by the DIEP flap at Hanoi Medical University Hospital from June 2019 to June 2021.

## 2. Patients and Methods

### 2.1. Patients

This is a prospective study of 30 breast cancer patients who underwent intermediated breast reconstruction with DIEP flap after mastectomy from June 2019 to June 2021. They were selected based on the following inclusion criteria: (1) who were residents in the Hanoi Medical University Hospital, (2) who wrote an informed consent to participate in the study, (3) who were 18 years of age and above, and (4) who underwent immediate breast reconstruction for DIEP flap after mastectomy.

The patients were selected as candidates for immediate reconstruction under evaluation by both the oncological and plastic surgeon in the outpatient clinic. Patients with metastatic disease and evidence of spread beyond local disease were excluded for immediate reconstruction.

Since this study focused on immediate unilateral breast reconstruction only, delayed or bilateral reconstructions were excluded. Other exclusion criteria included: (1) history of previous esthetic or reconstructive breast surgery, (2) contralateral breast surgery, (3) cancer recurrence, and (4) psychiatric disorder.

Complications including fat necrosis, partial flap loss, total flap loss, venous congestion, venous occlusion, breast seroma, breast hematoma, abdominal hernia, and medical complications were reported. Study data were collected and managed using SPSS 20.0. Frequencies and proportions were used to present categories variables. Descriptive statistics were calculated for all variables. Variables associated with breast complication were analyzed by using chi-square and Fisher's exact tests corrected for continuity. Mann–Whitney's tests were used to investigate the relationship between BMI scores and the occurrence of complications. All tests were two-sided and *p* values below 0.05 were considered as statistically significant.

The patients evaluated a study by answering specific questionnaire with the answers rating from 1 (unsatisfied) to 4 (very satisfied) regarding their satisfaction of reconstructed breast and satisfaction of the overall operation procedure. All patients were contact by phone and scheduled to return for a follow-up visit after 1 month and 3 months.

### 2.2. Surgery

Before surgery, all patients undergo preoperative perforator mapping and measurement of the diameter vessels using ultrasonography. The Doppler flowmetry of the epigastric artery on the abdominal wall was done routinely in all patients before and after the breast reconstruction, which provides important information concerning the location, caliber, and flow in the perforating vessels. General anesthesia was administered to the patients. Two teams of oncological and plastic surgeons joined simultaneously to reduce the operation time. If the tumor had a low rate of local recurrence (i.e., early-stage, node-negative, and HER2-negative) and the edge of the tumor was 2 cm away from the nipple, we would perform NSM in case the subareolar intraoperative frozen section was negative. In our institution, because of the lacking radioactive colloid, we could not perform sentinel lymph node biopsy (SLNB) routinely in early-stage breast cancer patients; therefore, we choose to perform axillary lymph node dissection and mastectomy in node-negative breast cancer patients to avoid the probable false-negative results of SLNB with single blue dye technique.

Meanwhile, the plastic team elevated the flap and prepared the skin pocket on the chest wall, recipient vessels in the axilla or chest wall. The skin and subcutaneous tissue island were lifted off the external oblique fascia until the perforator was encountered. Once the preferred perforators were identified, the anterior rectus was incised around the neurovascular bundle. The incision in the fascia was prolonged laterally and distally towards the groin to facilitate the dissection of the main stem of the inferior epigastric vessels. Then, the rectus abdominis muscle was split longitudinally and inferiorly to expose the desired pedicle length. Branches of the intercostal nerves that pass anterior to the deep inferior epigastric vessels should be preserved.

Afterward, the anterior rectus sheath was closed, and either the abdominoplasty or umbilicoplasty was performed. The flap was transferred to the thorax, placed at the region of the mastectomy, and sutured to the anterior chest wall. Thoracodorsal vessels were used in all the immediate reconstructions as the recipient's vessels, and a vascular microanastomosis was performed onto these vessels or onto the serratus branch. None of the patients received nerve repair. The flap was sutured to the pectoralis fascia to protect the pedicle.

## 3. Results

### 3.1. Perioperative Details

Clinicopathological characteristics are summarized in Table 1. Of all 30 patients, the mean age was 44.9 years old (range: 29-73). The mean tumor size measured by ultrasound was 1.98 cm. Twenty-eight patients (93.3%) were in stages I, and II, and two patients were stage III. Twenty-one patients (70%) received adjuvant chemotherapy. Five patients (16.7%) received neoadjuvant chemotherapy prior to immediate breast reconstruction, and five patients have completely responded.

### 3.2. Surgery

The overall flap success rate was 90%. The mean operating time was 341 minutes. All patients were treated by adjuvant therapy within 7 weeks postoperation, ranging from 21 to 49 days. Twenty patients (66.7%) had NSM. The mean postoperative hospital stay was 12.6 days. The number of perforators ranged was 1 to 2 with a mean of 1.30.

### 3.3. Complications

Twelve patients (40%) experienced a total of 16 complications from mild seroma to complete flap loss. Postoperative complication is split into three categories based on their site. In the breast complication group: four patients (13.3%) returned to the operating room due to venous congestions. Two patients (6.67%) had complete flap loss. Other complication: fat necrosis (6.7%), seroma (13.3%), and partial flap loss (3.3%). Two patients suffered abdominal wound dehiscence (6.7%). Systemic complications were pneumonia (3.3%) and pulmonary embolism (6.7%). No patients experienced abdominal hernias, hematoma, and infection donor/recipient site.

Higher incidences of breast complication were associated with one perforator group (38.1% vs. 22.2% in the two perforator groups); however, there was no statistical significance (*p* = 0.675) (Table 2).

After one-month postoperation, 88.9% of patients were satisfied with their breasts, and 74.07% were satisfied with the operation. After three months postoperation, 70.37% of patients were satisfied with their breasts, and 74.07% were satisfied with the operation (Table 3).

## 4. Discussion

Since DIEP flap was initially reported in 1999, this technique has been the standard treatment for breast reconstruction in breast cancer patients [10]. Immediate breast reconstruction yields better aesthetic results and reduce psychological distress than delayed reconstruction [6]. The Southeast Asian population is generally shorter in stature with a lower BMI and smaller breast size than Caucasian population [11]. In this study, all patients were not a candidate for breast conservation therapy because of tumor size relative breast size, multicentric disease, and rejection of radiotherapy, so breast reconstruction after mastectomy was a carefully selected treatment. So, we perform study of immediate DIEP flap reconstruction to review the first experience of this technique in Vietnam, where the new technique has not been popularized yet.

Five patients (16.7%) received neoadjuvant chemotherapy, initially had stages II and III breast cancer, and then underwent mastectomy and immediate DIEP flap reconstruction 4 weeks after the end of chemotherapy. The most chemotherapy regimens were triplet chemotherapy consisting of cyclophosphamide, taxane, and anthracycline with or without HER2-targeted therapy. All patients received adjuvant therapy within 3 to 7 weeks including those who underwent reoperation to repair or remove flaps. This indicated that the surgery complications did not postpone the postoperative treatment. In a large study by Beugels et al., 326 patients were included who underwent immediate DIEP flap reconstruction, of which 48 patients (14.7%) received neoadjuvant chemotherapy. There was no statistically significant difference in the incidences of reconstructive complications with or without neoadjuvant chemotherapy [12].

Reconstruction methods can be divided into implant based and autologous tissue reconstruction. Breast implant is a round, flexible silicone shell filled with saline or silicone gel. A common complication in patients receiving radiation therapy after silicone implant reconstruction is the formation of constrictive fibrous capsules around the implant and limits skin elasticity and the ability to expand; thus, autologous tissue options are preferred over implant-based techniques if the patient has an indication for radiation therapy [13]. After surgery, 11 patients (36.67%) had received adjuvant regional radiotherapy recommended by NCCN guidelines since autologous breast reconstruction does not alter the indications for radiotherapy in breast cancer patients [14]. Furthermore, a meta-analysis found that autologous reconstruction brings higher satisfaction to the overall outcome and cosmetic results than implant based [15]. Most patients diagnosed with breast cancer are at an age when they have excess fat under abdomen. DIEP flap, which allows the transfer of the same tissue from the abdomen to the chest as TRAM flap but not the sacrifices of the rectus muscle, becomes a mainstay for the repair of mastectomy defects. However, there was still limited data about the experience and safety for immediate DIEP flap reconstruction in Vietnamese patients. This study was conducted with the aim to shed light on the safety of the procedure.

**Figure 1:**
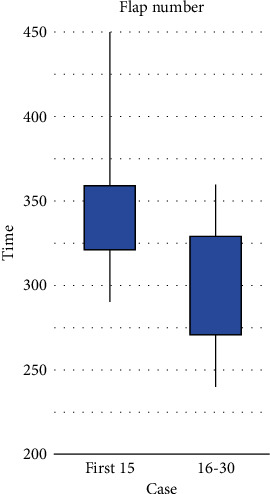
Comparison of two groups: operating time.

In our institute, after producing anesthesia, plastic surgeons dissected perforators, and oncologist surgeons perform mastectomy simultaneously to reduce the operating time. After reaching the plateau in the learning curve, the average operating time is markedly reduced from 367 minutes in the first 15 patients to 315 minutes in the last 15 patients (*p* = 0.019) (Figure 1). In an attempt to preserve the function of the rectus abdominis muscle, the surgeon must perform carefully to dissect small vessels, hence the prolonged operating time. The average reconstruction time was 341 minutes, which was consistent with the previous studies of Blondeel; one of the first people who had experience with DIEP flap reported in 1999 that the mean operating time was 372 minutes [10].

**Figure 2:**
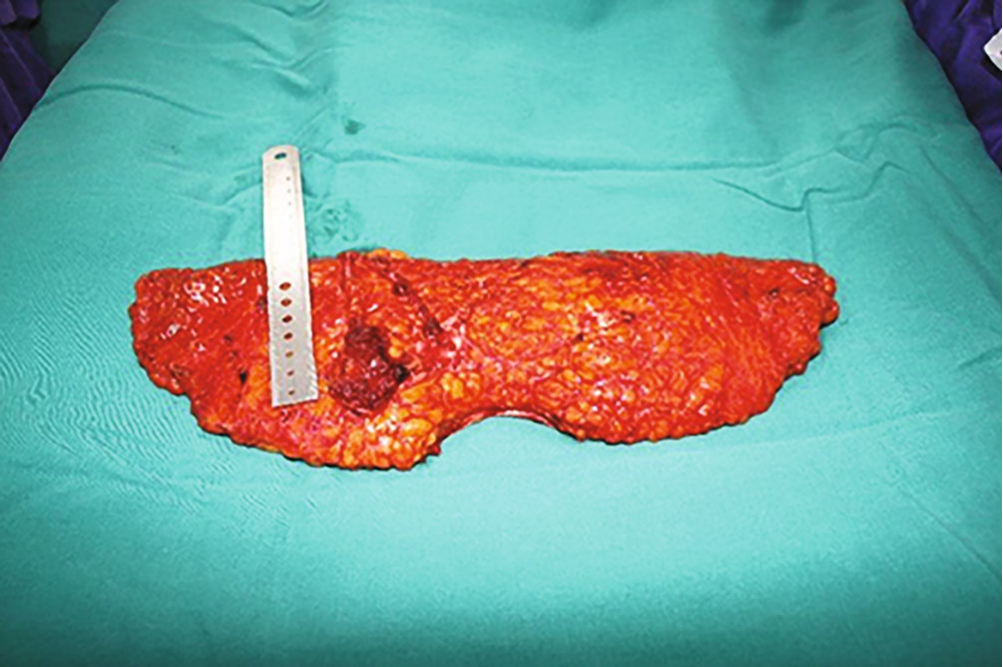
Example of 2 perforators for flap.

Of the 5 patients (16.7%) shown in Table 4 with reoperation, one (3.3%) had planned surgery to remove the distal part of the flap because of partial necrosis flap, and the other 4 patients (13.3%) had to have surgical emergencies due to venous congestion. After dissection, we regularly check the flow of anastomosis vascular with Doppler's ultrasound to detect vascular complications early. If venous congestion is recognized as a palpable fullness of the flap with a progressive blue/purple discoloration and brisk capillary refill of less than 1 second, secondary operation with augmentation of venous outflow is required. By adding a second deep venous anastomosis, venous outflow is significantly increased, and two patients was successfully revascularization. The two remaining flaps were still bleeding on the flap edge after repairing venous anastomosis and adding more perforator vein, so they undergo total flap removal. Notably, in these flaps, the deep venous system is sufficient for venous drainage before flap transfer, but the cross-sectional area of a single deep venous anastomosis is insufficient for adequate flap drainage after flap transfer and anastomosis. Venous congestion in these flaps is a result of convert venous hypertension from the deep to superficial system through valveless linking veins. In a retrospective review of 758 DIEP flaps, Gill et al. show that there is no correlation with the number of perforators and venous occlusion [16]. We usually use one thoracodorsal artery and one or two accompanying veins (Figure 2). Therefore, these data support that one perforator is as effective as more perforators in terms of blood supply. Previous studies have shown that increasing the number of perforators increases the incidence of complications [16]. To overcome this problem, much effort has been made; drainage by additional anastomosis of the superficial inferior epigastric vein (SIEV) is one of them. However, we need further prospective or randomized control trials to support its use to reduce the risk of venous congestion. The rate of reoperation has been decreasing, owing to experience gaining over time, especially that rate fell from 15% in initial reports to 2.6% in recent reports in high-volume centers where more than 40 DIEP flaps were performed per year [17–19]. Along with an accumulation of cases, proper education and training would help surgeons and centers with less experience perform breast reconstruction safely and reduce the learning curve period.

Three patients (10%) with fat necrosis, all of them, did not require intervention. Breast fat necrosis is a reaction caused by the disruption of vessel, and it can be differentially diagnosed from local recurrence by biopsy. There is no widely definition of fat necrosis in DIEP flap. This complication based on clinical presentation including: absolute size (inconsistent cut off in different authors) detected clinically or by ultrasound and confirmed not to be a malignant recurrence [20]. Fat necrosis rates ranged from 12 to 45% on clinical examination with the highest incidence in smoking, postreconstruction radiotherapy [21]. This complication is usually mild and self-limited, but clinicians should distinguish it from local recurrence on certain cases.

Due to the sacrifice of the rectus abdominis, the TRAM flap is associated with high risk of abdominal hernia, which was independent of the type of TRAM flap used. Moreover, Blondeel et al. compared patients undergoing unilateral free TRAM versus unilateral DIEP flaps and found a statically significant improvement in trunk flexing, upper trunk rotational strength, and rate of abdominal hernia in the DIEP group [22]. These data indicated that DIEP flap had more advantages than free TRAM flap on complication of the donor site, but patients who had previous abdominal surgery make scar tissue throughout the lower rectus abdominis muscle hard to find and dissect of perforator and vessel.

The mean hospital stays in our study were longer than in other centers (12.6 vs. 8.45 days) [18]. It can be distributed to the difference in structure of outpatient care between the healthcare systems. In developed countries, patients could be discharged soon after surgery and continue following up at outpatient clinics, which lacking in many developing countries like Vietnam.

**Figure 3:**
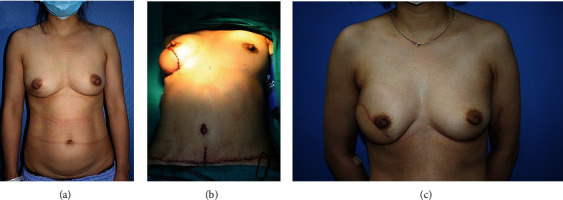
The preoperative (a), operation day (b), and 6-week postoperative (c) view of a 45-year old woman after immediate DIEP flap breast reconstruction.

Each perforator of the DIEP flap is more sophisticated than the free TRAM flap, which means comparable aesthetic results can be achieved with both flaps. Because of its long vascular pedicle and central location of the microsurgical anastomosis, the flap is flexible to match the shape of the patient's breast (Figure 3). But the DIEP or the other free flap incurs higher hospital total charges and has longer lengths of stay compared with pedicle TRAM [17]. Despite immediate reconstruction, patients who underwent DIEP flap reconstruction still had second chance to improve outcome by additional procedures including nipple reconstruction, scar revision, and volume adaptation to achieve the best symmetry 6 months later.

Limitations of this study include single center and relatively sample size. Further studies need to be conducted with more centers involved large sample sizes to overcome this limitation. However, the study still has its own value since this is the initial experience of an advantage flap of autologous breast reconstruction, which yields better aesthetic results and reduces psychological distress as a treatment of choice for many breast cancer patients.

The evidence suggests that DIEP flap has become the preferred choice for immediate breast reconstruction, for better cosmesis can be achieved with skin and soft tissue. In certain situations, such as the case with inadequate abdominal tissue to replace the breast or a very dominant superficial venous system, we had success with the thoracodorsal artery perforator flap as an alternative.

## 5. Conclusion

Breast mounds reconstructed with the patient's skin and fat behave naturally, achieving the consistency of native breast. Because of the advantage of the DIEP flap for immediate breast reconstruction, we have adopted this method as our standard of care for breast cancer. As microsurgical experience increases, we think that the benefits of this flap technique will outweigh its risk.

## Figures and Tables

**Table 1:** Clinicopathological characteristics.

Characteristics	Value
Mean age (years)	44.9 (range, 29–73)
Mean BMI	21.45 (range, 18.73–25.66)
Tumor size (cm)	1.98 (range, 0.7–6.0)
Tumor	
T1	17 (56.7%)
T2	11 (36.6%)
T3	2 (6.7%)
TNM stage	
Stage I	17 (56.7%)
Stage II	9 (30%)
Stage III	4 (13.3%)
Mean operative time (hours)	341.33 (range, 240-520)
Meantime to receive adjuvant systemic therapy (days)	34.68 (range, 21-49)
Neoadjuvant chemotherapy	5 (16.7%)
Adjuvant chemotherapy	21 (70%)
Complete responded	4 (80%)
Partial responded	1 (20%)
NSM	20 (66.7%)
Mean postoperative hospital stay (days)	12.60 (range, 6–18)
Number of perforators	1.30 ± 0.47

**Table 2:** Variables associated with breast complications.

		Breast complications		*p*
		No	Yes	
Neoadjuvant chemotherapy	Yes	4 (80%)	1 (20%)	0.64^∗^
	No	16 (64%)	9 (36%)	
The number of perforators	One	13 (61.9%)	8 (38.1%)	0.675^∗^
	Two	7 (77.8%)	2 (22.2%)	
Microsurgical anastomosis	Thoracodorsal artery	13 (65%)	7 (35%)	1.0^∗^
	Internal mammary artery	7 (70%)	3 (30%)	
Mean BMI		21.22 ± 1.78	21.94 ± 0.93	0.113^+^

^∗^Fisher's exact test. ^+^Mann–Whitney test.

**Table 3:** Satisfaction with breast.

	Breast		Overall operation	
	One month postoperation	Three months postoperation	One month postoperation	Three months postoperation
Unsatisfied	1 (3.7%)	0	1 (3.7%)	0
Neutral	3 (11.11%)	7 (25.93%)	7 (25.93%)	6 (22.22%)
Satisfied	10 (37.04%)	9 (33.33%)	4 (14.81%)	6 (22.22%)
Very satisfied	14 (51.85%)	10 (37.04%)	16 (59.26%)	14 (51.85%)

**Table 4:** Operative complications.

Complications	Incidence (*n*%)
Breast complications	
Venous congestion	4 (13.3%)
Seroma	4 (13.3%)
Fat necrosis	3 (10%)
Abdominal complications	
Wound dehiscence	2 (6.7%)
Abdominal hernia	0
Systemic complications	
Pulmonary embolism	2 (6.7%)
Pneumonia	1 (3.3%)
Hematoma	0

## Data Availability

All data underlying the results are available as part of the article, and no additional source data are required. All data sources described in this study are directed at the corresponding author.
